# Detection and correction of patient motion in dynamic ^15^O-water PET MPI

**DOI:** 10.1007/s12350-023-03358-5

**Published:** 2023-08-28

**Authors:** Nana L. Christensen, Jonny Nordström, Simon Madsen, Michael A. Madsen, Lars C. Gormsen, Tanja Kero, Mark Lubberink, Lars P. Tolbod

**Affiliations:** 1 Department of Clinical Medicine, Aarhus University, Nordre Ringgade 1, 8000 Aarhus C, Denmark; 2 Department of Nuclear Medicine & PET, Aarhus University Hospital, Aarhus N, Denmark; 3 Centre for Research & Development, Uppsala/Gävleborg County, Gävle, Sweden; 4 Department of Surgical Sciences/Nuclear Medicine & PET, Uppsala University, Uppsala, Sweden; 5 Medical Imaging Centre, Uppsala University Hospital, Uppsala, Sweden

**Keywords:** PET, myocardial blood flow, image analysis, perfusion agents

## Abstract

**Background:**

Patient motion constitutes a limitation to ^15^O-water cardiac PET imaging. We examined the ability of image readers to detect and correct patient motion using simulated motion data and clinical patient scans.

**Methods:**

Simulated data consisting of 16 motions applied to 10 motion-free scans were motion corrected using two approaches, pre-analysis and post-analysis for motion identification. Both approaches employed a manual frame-by-frame correction method. In addition, a clinical cohort was analyzed for assessment of prevalence and effect of motion and motion correction.

**Results:**

Motion correction was performed on 94% (pre-analysis) and 64% (post-analysis) of the scans. Large motion artifacts were corrected in 91% (pre-analysis) and 74% (post-analysis) of scans. Artifacts in MBF were reduced in 56% (pre-analysis) and 58% (post-analysis) of the scans. The prevalence of motion in the clinical patient cohort (n = 762) was 10%. Motion correction altered exam interpretation in only 10 (1.3%) clinical patient exams.

**Conclusion:**

Frame-by-frame motion correction after visual inspection is useful in reducing motion artifacts in cardiac ^15^O-water PET. Reviewing the initial results (parametric images and polar maps) as part of the motion correction process, reduced erroneous corrections in motion-free scans. In a large clinical cohort, the impact of motion correction was limited to few patients.

**Supplementary Information:**

The online version contains supplementary material available at 10.1007/s12350-023-03358-5.

## Introduction

Commonly used perfusion tracers in clinical cardiac PET imaging are ^13^N-ammonia, ^82^Rb-chloride, and ^15^O-water,^1^ where the latter is the gold standard for non-invasive quantification of myocardial blood flow (MBF).^2^ ^15^O-water possesses characteristics making it an ideal flow tracer; it is freely diffusible and metabolically inert with almost 100% extraction. However, it is not retained in the myocardium and thus, late-uptake static images cannot be used. Instead, interpretation is based on the assessment of quantitative MBF, and parametric images derived from a 1-tissue compartmental model using a dynamic image series.^1,3^

Patient motion is a limiting factor for both static and dynamic myocardial perfusion imaging (MPI) leading to poor image quality and erroneous quantitation.^4,5^ Previous studies on patient motion have reported a high occurrence of motion, with 30-68% of image acquisitions affected.^6–10^ Motion can be random with independent patterns of motion magnitude and direction or due to coughing.^11,12^ However, the most common causes of patient motion are related to discomfort from pharmacological stress agents, and the associated changes in respiratory pattern, which may result in a continuous displacement of the mean position of the heart,^13^ or even rapid displacements which may appear like random motion.^6,8^ In dynamic imaging, cardiac creep and motion in the early blood phase, has a significant impact on quantification, particularly in the right coronary artery (RCA) territory, and can cause severe errors in the estimation of MBF up to 500%.^7,8,14^

Several motion correction methods have been proposed ranging from manual or semi-automatic frame-by-frame realignment, gating,^7,14^ or motion tracking using external devices^15,16^ to automatic methods.^10,17–20^ Most automatic methods and methods using external devices have been limited to late static imaging; however, newer list-mode-based methods may be promising for dynamic data as well.^21^ Unfortunately, list-mode processing tools need to be integrated into the PET/CT scanner hardware and are not yet commercially available for dynamic acquisition. Manual or semi-automatic frame-by-frame realignment is available in several commercial cardiac software packages for use with retention tracers, but, currently, no tools are available for ^15^O-water.

In contrast to other MPI tracers, quantitative MBF values from ^15^O-water are based on tracer wash out rather than tracer uptake leading to a different sensitivity toward partial volume effects and patient motion. A recent study by Nordström et al. found only minor impact from PET/CT misalignment on the accuracy of MBF measurements using ^15^O-water PET, suggesting that PET/CT misalignment, i.e., inter-scan patient motion, is less problematic for ^15^O-water than for other tracers.^22^ Similarly, they found typical effects on MBF from different types of dynamic patient motion during the PET scan to be only slightly larger than the inter-observer variability.^6^ Nevertheless, some types of motion lead to large errors in the LAD and RCA territories, potentially affecting patient management.

In the present study, we present a tool for manual frame-by-frame motion correction of ^15^O-water MPI, and, using previously published simulated motion data,^6^ we evaluated the ability of image readers to visually identify and correct patient motion using two different approaches. In addition, a large cohort (n = 762) of clinical ^15^O-water scans were included for investigation of prevalence and effect of motion and motion correction.

## Methods

### Simulated motion study

In this part of the study, we investigated the ability to visually detect and manually correct patient motion. Data consisted of myocardial stress ^15^O-water PET scans with simulated motion from Nordström et al.^6^ Of the original 17 simulated motions, five motions with low amplitude were omitted, and instead, 4 new motions were added. Thus, this study included a data set consisting of 16 different simulated motions with an amplitude of 5 to 20 mm applied to data from 10 motion-free clinical scans, yielding 160 simulated scans. The 10 motion-free scans were randomly selected after motion was ruled out by visual inspection.^6^ The motions are summarized in Table S1 (Supplementary information). The simulated patient motions were divided into four different types, representing typical patterns of patient motion. The first type was simulated as a 10 or 20-mm displacement in the anterior (+ *y*) and cranial (+ *z*) direction, resembling a patient’s reaction to a stress agent (Stress Agent 1 to 4). In the second type, the motion was a 10 or 20-mm linear slide in the caudal (− *z*) direction either throughout the scan or from 1-minute post-injection until the end of the scan (Linear Slide 1 to 4). The third type was simulated as a 10 or 20-mm anterior displacement (+ *y*) of a single frame at the peak of the myocardial time-activity-curve (TAC) and after 1 and 2 minutes, simulating motion from a cough (Peak Cough 1 to 2 and Late Cough 1 to 4). In the fourth type, the effects of cardiac creep were simulated by a 5 or 10-mm linear slide in the cranial direction (+ *z*) from 1-minute post-injection until the end of the scan (Cardiac Creep 1 to 2).

All simulated motion scans were motion corrected in a blinded fashion. Two different approaches to identify motion were examined: (1) a pre-analysis approach, where motion was assessed in the raw dynamic PET images, and, (2) a post-analysis approach, where derived parametric images and polar maps were assessed for motion artifacts, leading to a review of the raw dynamic PET images only in scans with suspected motion. In both approaches, motion correction was performed using the same manual frame-by-frame method. Scans were analyzed without simulated motion (motion-free), with simulated motion (pre-correction) and after motion correction (post-correction) with comparison of global and regional MBF.

### Clinical patient study

All patients enrolled in the ^15^O-H_2_O PET Cohort study (NCT04451551) at Aarhus University Hospital between August 2020 and January 2022 were included (n = 1545). The patients were referred for a ^15^O-water MPI scan on clinical indication (evident or suspected ischemic heart disease). We searched the clinical reports for the terms ‘motion,’ ‘inconclusive,’ and ‘cannot be estimated.’ If ‘motion’ was used in connection with ‘inconclusive’ or ‘cannot be estimated,’ the exam was categorized as ‘inconclusive due to motion.’ If ‘motion’ was used alone, the exam was categorized as ‘conclusive with reservations due to motion.’ The motion correction tool became available to clinicians halfway through the period (April 2021). For patients examined after this date (n = 762), analyses, where the motion correction tool was utilized, were collected, and stress MBF for the three coronary territories pre- and post-motion correction was compared. Motion correction in the clinical patient study corresponded to the post-analysis approach.

### Motion correction

**Figure 1:**
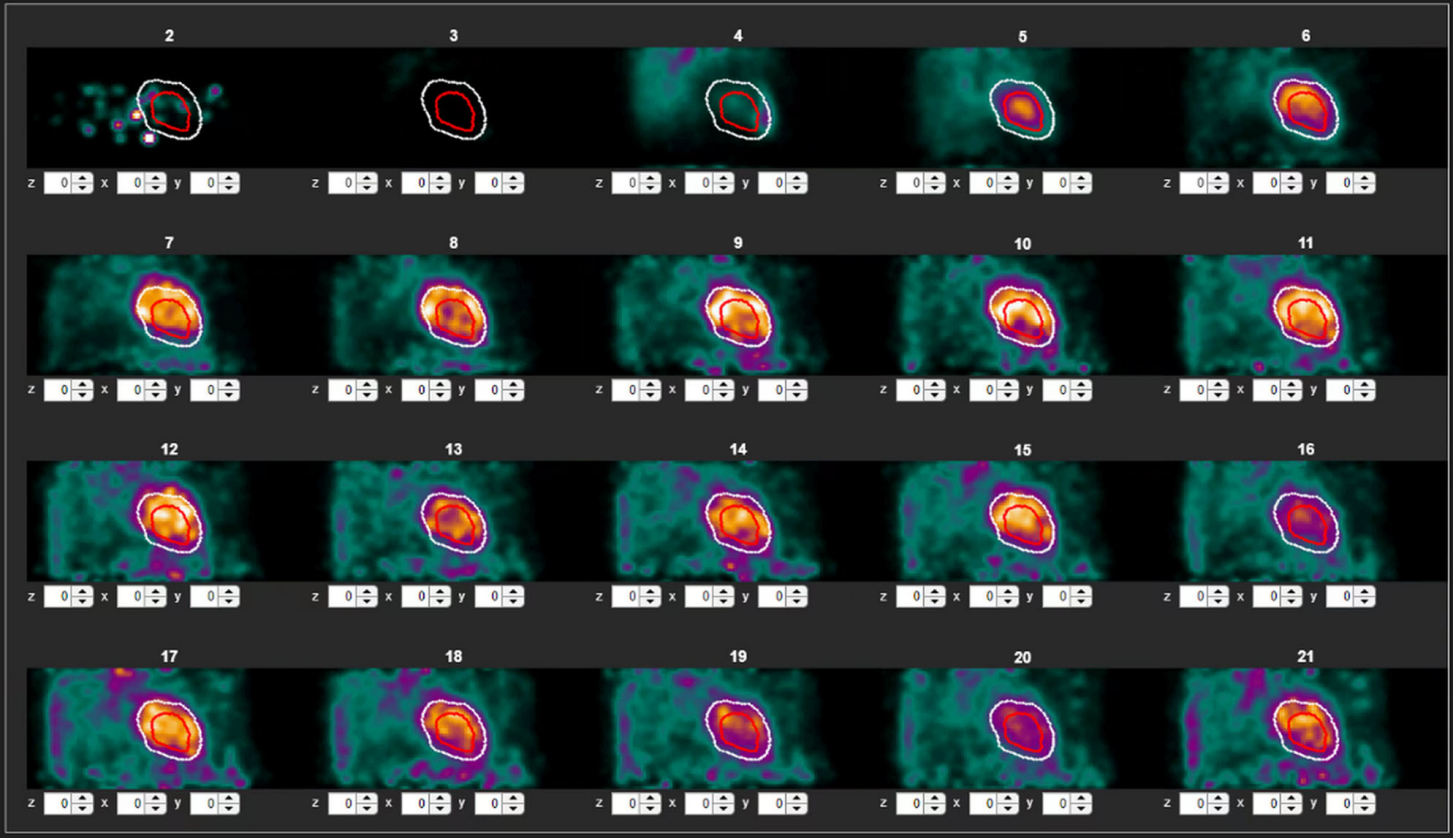
Frame-by-frame motion correction software. All 21 frames are displayed, showing an overlay of the left ventricular segmentation from the aQuant pre-correction analysis. Each frame can be manually shifted in the *x*-, *y*-, or *z*-direction

All simulated motion scans and exams from the clinical patient cohort containing motion were analyzed pre- and post-correction using aQuant (MedTrace Pharma A/S, Hørsholm, Denmark). Motion correction was performed manually frame-by-frame using an in-house built tool with a graphical user interface in Matlab (R2020b, The MathWorks Inc., Natick, Massachusetts, USA) (Figure 1). In the interface, all frames were displayed with an overlay of the LV wall segmentation from the initial aQuant analysis. The user was able to scroll through the image volume and switch between sagittal and coronal displays. The interface allowed the user to shift each frame independently in the *x*- (right/left), *y*- (anterior/posterior), or *z*-direction (cranial/caudal) in steps of 1 mm with an immediate update of the display. Shifting of frame image volumes was performed from the original frame using a single rigid 3D transformation to avoid the effects of repeated transformations. In the interface, side-by-side replays of the dynamic frames pre- and post-correction could be viewed to assess the correction.

MBF for each coronary territory and global values both pre- and post-correction were reported. Myocardial perfusion was considered normal at MBF_stress_ values ≥ 2.3 mL⋅min^−1^⋅g^−1^.^2^ Scans with large motion artifacts were defined as having > 20% deviation in MBF in one or more coronary territories compared to the motion-free scan.

### PET acquisition

The data acquisition of the simulated motion study is described elsewhere.^6^ In the clinical study, all exams were performed using a GE Discovery MI DR PET/CT system (GE Healthcare, Milwaukee, Wisconsin, USA). All patients were asked to abstain from caffeine for a minimum of 24 hours before the examinations. The protocol consisted of an ultra-low-dose CT scan for attenuation correction followed by a 4-minute dynamic rest and stress PET acquisition. Patients received an intravenous bolus injection of 400 MBq ^15^O-water using the MedTrace MT100-P3 automated ^15^O-water production and delivery system (MedTrace Pharma A/S, Hørsholm, Denmark). All stress exams were performed during pharmacological stress using adenosine with an infusion rate of 0.140 mg⋅kg^−1^⋅min^−1^ for 6 minutes. After 2 minutes of adenosine infusion, the ^15^O-water injection and the PET image acquisition were started simultaneously. The dynamic PET data was divided into 21 time-frames with a frame structure of 10 × 5s, 4 × 10s, 2 × 15s, 3 × 20s, and 2 × 30s. All images were reconstructed to 3.27 × 3.27 × 3.27 mm^3^ voxels using a time-of-flight ordered-subset expectation maximization algorithm with all corrections applied.

### Image analysis

Using aQuant, MBF values were calculated for three datasets: ‘MBF motion-free,’ ‘MBF uncorrected,’ and ‘MBF motion-corrected.’ ‘MBF motion-free’ refers to MBF calculated from the original images without simulated motion. ‘MBF uncorrected’ refers to MBF calculated from the images with simulated motion. ‘MBF motion-corrected’ refers to MBF calculated from the images after motion correction. To quantify the effects of motion on MBF, the relative deviation in MBF was defined as the percentage difference in MBF between the motion-free images and (1) the images with motion artifacts and (2) the motion-corrected images. MBF artifacts were defined as deviations in MBF caused by motion and were quantified as the percentage of images where MBF artifacts were reduced after motion correction compared to the uncorrected images.

Dichotomous clinical interpretation of the scans were performed using cut-off for MBF_stress_ of 2.3 mL⋅min^−1^⋅g^−1^ globally or in one or more coronary territory.^2^

### Statistical analysis

Data normality was assessed using Shapiro–Wilk test. A Wilcoxon signed-rank test was used to assess the differences in MBF between motion-free data and data with simulated motion, both pre- and post-motion correction. Data are presented as heatmaps of median relative deviation (%), maximum relative deviation (%), and significance-values of the signed-rank test (without correction for multiple comparisons). *P* < .05 was considered significant. The impact of motion correction on clinical data was assessed by Bland–Altman plots comparing MBF_stress_ pre- and post-correction, displaying absolute median differences and 95% limits of agreement. Bland–Altman plots comparing MBFstress pre- and post-correction, displaying absolute median differences and 95% limits of agreement assessed the impact of motion correction on clinical data.

## Results

### Simulated motion study

The median global MBF for the 10 original motion-free scans was 2.35 (IQR: 0.82) mL⋅min^−1^⋅g^−1^. At a territorial level, LAD had a median MBF of 2.27 (IQR: 1.27) mL⋅min^−1^⋅g^−1^, while RCA had a median MBF of 2.30 (IQR: 0.73) mL⋅min^−1^⋅g^−1^. In LCx the median MBF was 2.31 (IQR: 1.10) mL⋅min^−1^⋅g^−1^.

The results from the two motion correction approaches are summarized in Table 1. Of the 170 scans, 94% were motion corrected when using the pre-analysis approach to identify motion. The corrections were made in the *x*-, *y*-, and *z*-directions in 32%, 47%, and 95%, respectively. Displacements of > 10 mm in one or more frames were corrected in 16% of the images, with 7% corrected in the *x*-direction and 52% and 41% corrected in the *y*- and *z*-directions, respectively. Using the post-analysis approach, motion was detected and attempted corrected in 64% of the 170 scans. The corrections were made in the *x*-, *y*-, and *z*-directions in 1%, 44%, and 69%, respectively. Displacements of > 10 mm in one or more frames were introduced in 23% of the scans, with 72% and 28% corrected in the *y*- and *z*-directions, respectively, and no large corrections made in the *x*-direction.

**Table 1:** Results from the simulated motion study

	Pre-analysis approach	Post-analysis approach
Total corrected scans (%)	94	64
Correction in *x*-direction (%)	32	1
Correction in *y*-direction (%)	47	44
Correction in *z*-direction (%)	95	69
Correction of images with displacements > 10 mm (%)	16	23
Correction in *x*-direction for images with displacements > 10 mm (%)	7	0
Correction in *y*-direction for images with displacements > 10 mm (%)	52	72
Correction in *z*-direction for images with displacements > 10 mm (%)	41	28
Territories with reduced MBF artifacts (%)	54	57
Correction in the 10 motion-free scans (#)	8	1

Pre-analysis approach: Motion assessed using dynamic PET images only. Post-analysis approach: Motion assessed using dynamic PET images, pre-motion-correction parametric images, and polar maps

**Figure 2:**
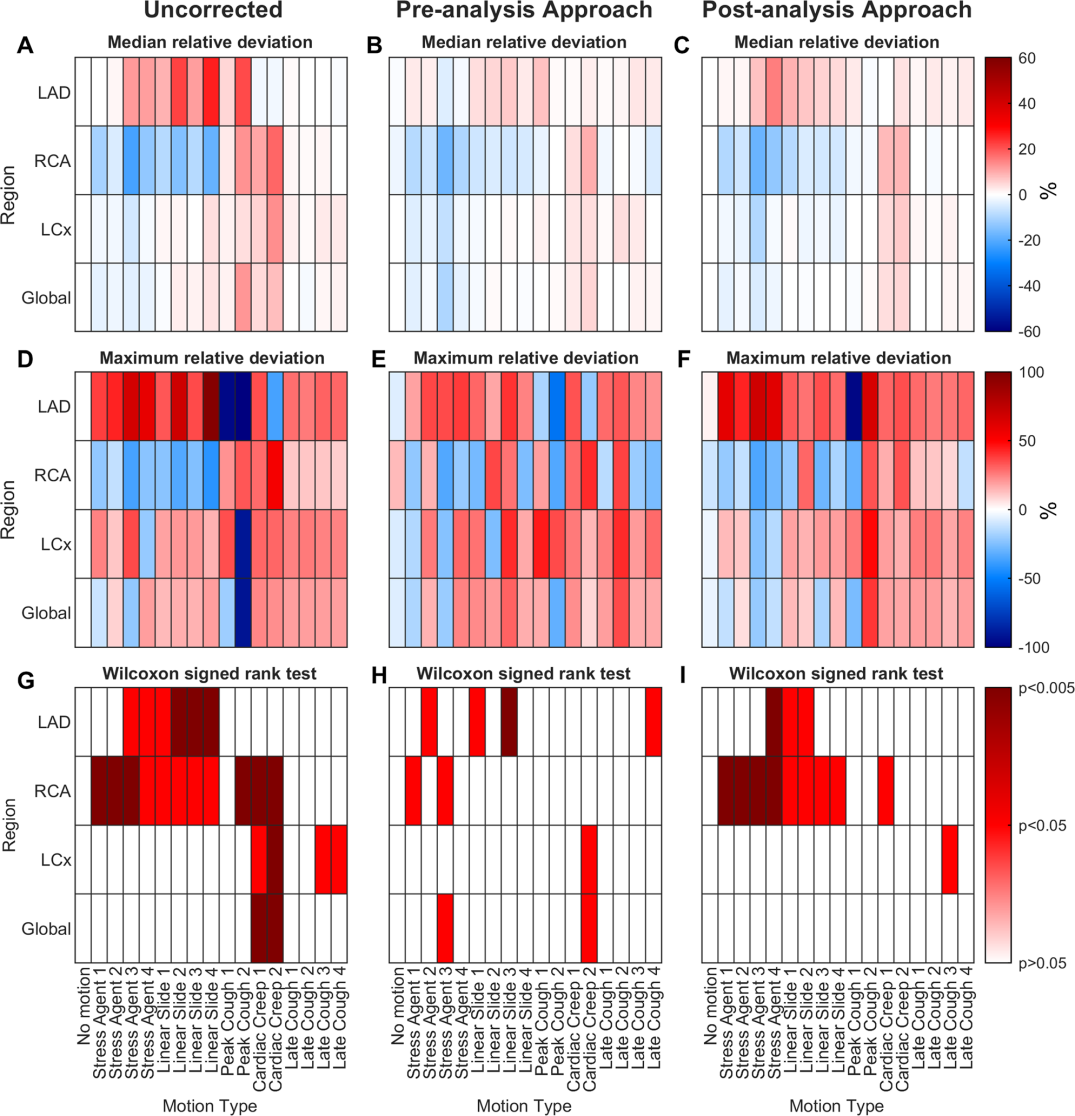
Heat maps of uncorrected (**A**, **D**, **G**) and motion corrected images using the pre-analysis (**B**, **E**, **H**) and post-analysis (**C**, **F**, **I**) approach. The *X*-axis represents each type of motion. The *Y*-axis represents the coronary territories (LAD, RCA, LCx, and global). Median relative deviation (%) in MBF from the original motion-free images in uncorrected (**A**) and motion-corrected (**B**) images. Maximum relative deviation (%) in MBF from the original motion-free images in uncorrected (**D**) and in motion-corrected images using the preanalysis approach (**E**) and post-analysis approach (**F**). Results of the Wilcoxon signed-rank test, showing level of significance (*P* > .05, *P* < .05 or *P* < .005) in MBF deviation from the original motion-free images in uncorrected (**G**) and in motion-corrected images using the pre-analysis approach (**H**) and post-analysis approach (**I**). Every cell in **A**, **B**, **C**, **G**, **H** and **I** contains results from all 10 patients. Every cell in **D**, **E** and **F** contains results from the one patient with the highest maximum deviation

In scans with large motion artifacts, motion correction was performed in 91% using the pre-analysis approach and 74% using the post-analysis approach. Overall, motion correction reduced artifacts partially in 65% of the scans reviewed using the pre-analysis approach (corresponding to 54% of all territories) and in 72% of the scans reviewed using the post-analysis approach (corresponding to 57% of all territories). In the 10 motion-free scans, the pre-analysis approach resulted in incorrect identification of motion in eight scans, whereas the post-analysis approach led to motion being falsely identified in five scans. The median deviation in MBF introduced by erroneous correction was − 1.2% (max 13%). Deviation in MBF from the motion-free scans was reduced in 84% (pre-analysis approach) and 82% (post-analysis approach) of the territories with large motion artifacts (median relative reduction 46%, 95% percentile range − 16% to 96%). The median relative deviation in MBF from the motion-free scans is presented in Figure 2A. The heat maps of the uncorrected images show that MBF in the LAD and RCA territories were most affected by motion artifacts, resulting in increased median MBF in LAD and decreased median MBF in RCA in some of the motion types. The effect was less pronounced in the LCx territory and globally. Across all regions, every motion type except the late coughs affects the median relative deviation in MBF. Following motion correction, both approaches reduced the median relative deviation in MBF in LAD and to a lesser degree in RCA (Figures 2B, C). None of the median relative deviations in MBF were increased by motion correction. In the uncorrected images, the greatest maximum relative deviation in MBF was observed in the LAD territory as an overestimation of MBF (Figure 2D). Negative maximum deviations were observed for several motion types, seen as an underestimation of MBF, especially in RCA. Motion correction using both approaches reduced the largest maximum relative deviations; however, deviations larger than ± 50% were still found (Figure 2E, F). The maximum relative deviation increased in several types of motion in all regions after motion correction. Using the pre-analysis approach, the maximum relative deviation increased by more than 10 percentage points in one or more coronary territories in 35% of the motions, and more than 20 percentage points in 12% of the motions. For the post-analysis approach, these numbers were much smaller as only 6% of the motions increased by more than 20 percentage points in maximum relative deviation. A Wilcoxon signed-rank test was conducted to compare MBF deviations from the motion-free images in uncorrected images (Figure 2G) and motion-corrected images using the pre-analysis approach (Figure 2H) and post-analysis approach (Figure 2E). In the uncorrected images, a significant deviation in global MBF was observed for two motion types. A total of 13 motions led to significant deviations in MBF globally, or in one or more coronary territories. After motion correction, the number of motions with significant deviations in MBF were reduced for both approaches (7 and 10 for pre- and post-analysis, respectively).

**Figure 3:**
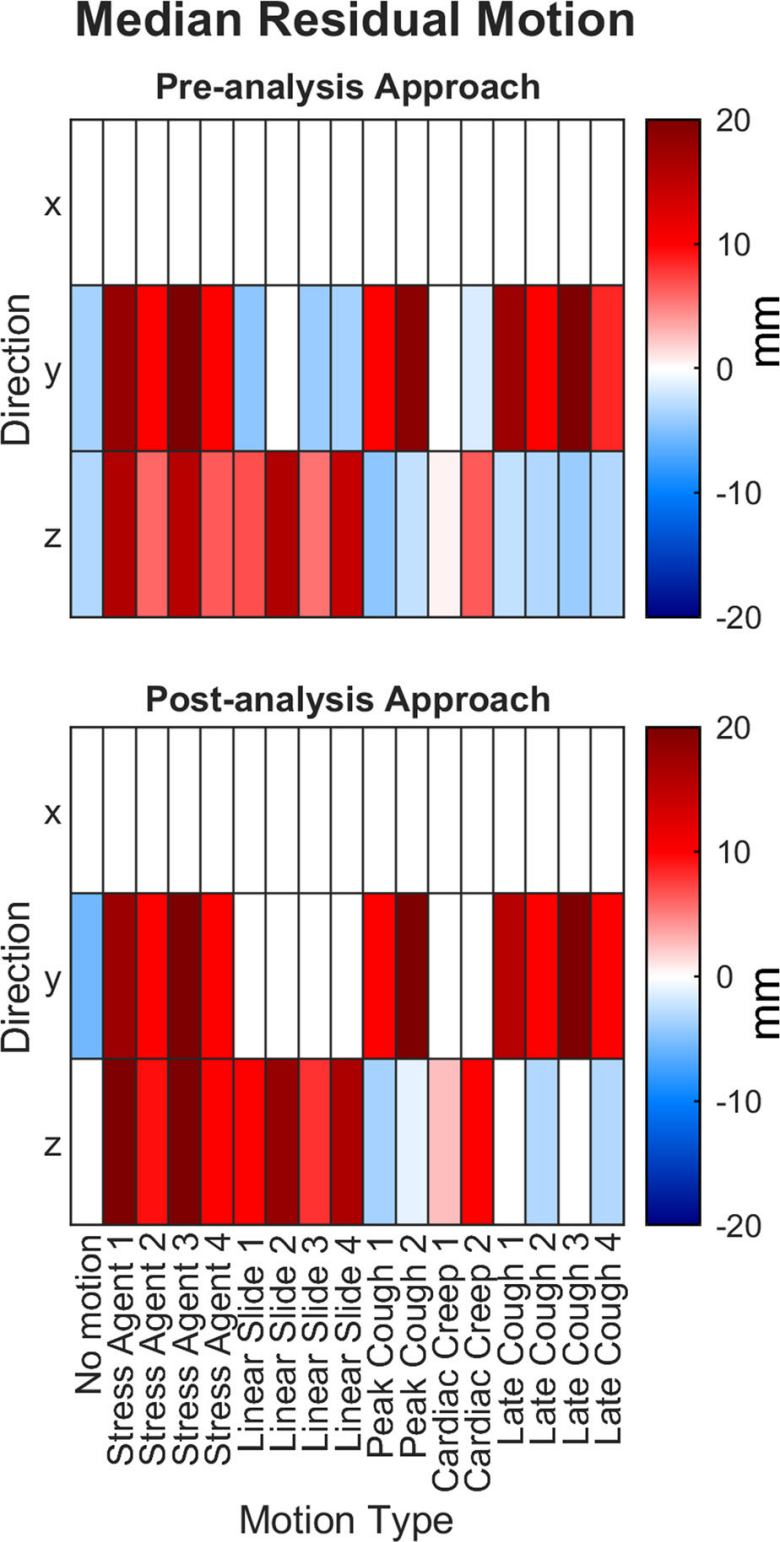
Median residual motion calculated as the difference between the actual simulated motion and the motion corrected by the two motion correction approaches. Positive values indicate under-correction, while negative values indicate over-correction. The values represent the magnitude of the residual motion in millimeters

To relate the motion correction applied using the two approaches to the actual simulated motion, the degree of correction was assessed by calculating the median residual motion, as illustrated in Figure 3. The median residual motion represents the difference between the actual simulated motion and the motion corrected using the two approaches, with a positive value indicating undercorrection and a negative value indicating overcorrection. Both approaches tended to underestimate the motion in the *y*- and *z*-directions for most motion types. Interestingly, the same types of motion were found to be undercorrected regardless of the approach used.

The impact of motion correction on the interpretation of the 170 simulated motion images was evaluated and summarized in Table 2 and presented as heat maps in Figure S1 (supplementary information). The table presents the number of examinations where the analysis changed from positive (MBF < 2.3 mL⋅min^−1^⋅g^−1^) to negative (MBF ≥ 2.3 mL⋅min^−1^⋅g^−1^) or from negative to positive globally or in one or more territories following motion correction. The pre-analysis approach resulted in a change of interpretation in 41% of cases, while the post-analysis approach led to a change in 27% of cases. However, in both scenarios, only 50% of these changes were accurate.

**Table 2:** The impact of motion correction on the interpretation of cardiac ^15^O-water PET results in 170 scans with simulated motion, based on a 2.3 mL⋅min^−1^⋅g^−1^ threshold for MBF_stress_

		Pre-analysis approach		Post-analysis approach	
		Changed (#)	True (#)	Changed (#)	True (#)
Global	Positive to negative	5	4	2	2
	Negative to positive	17	2	15	4
LAD	Positive to negative	5	5	5	5
	Negative to positive	11	10	12	10
RCA	Positive to negative	14	4	2	2
	Negative to positive	24	4	16	3
LCx	Positive to negative	9	5	2	1
	Negative to positive	15	6	12	4
Any	Positive to negative	29	15	8	7
	Negative to positive	40	18	38	16

Based on changes in MBF globally or in any of the coronary territories, motion correction using the pre-analysis approach altered the interpretation of 69 scans, while using the post-analysis approach, the interpretation was altered in 46. The change in interpretation was considered true, if the post-motion correction interpretation agreed with the interpretation of the original motion-free scan

### Clinical patient study

During the retrospective review period 1545 patients underwent a clinical ^15^O-water PET/CT exam. Fifty exams were repeated within 2 months (3.2%) of which 14 (0.91%) exams were categorized as ‘inconclusive due to motion.’ The remaining repeated scans may be due to suspected insufficient response to adenosine. An additional 50 exams (3.2%) were categorized as ‘conclusive with reservations due to motion.’ When the motion correction tool was available, 762 exams were conducted. Of these, the reporting nuclear medicine physician performed motion correction in 80 (10%) patients. In one exam, image artifacts caused by incorrect blood segmentation were wrongfully attributed to motion. Before motion correction, the mean global MBF_stress_ of the 80 exams was 2.51 ± 0.98 (LAD 2.51 ± 1.04, RCA 2.47 ± 1.04, LCx 2.62 ± 1.13) mL⋅min^−1^⋅g^−1^. In 44% of the exams, global MBF_stress_ was < 2.3 mL⋅min^−1^⋅g^−1^. In 14% of the exams, MBF_stress_ was < 2.3 mL⋅min^−1^⋅g^−1^ in only one coronary territory with the majority being RCA (10%). We observed high MBF_stress_ (> 5 mL⋅min^−1^⋅g^−1^) globally or in one coronary territory in 3.8% of the exams (six territories). Post-motion correction, the percentage of exams with reduced flow in only RCA dropped from 10% to 6%, and MBF_stress_ > 5 mL⋅min^−1^⋅g^−1^ was only observed in a single territory (change from 3.8% to 1.3%).

The effect of motion correction on the interpretation of patient exams was evaluated and presented in Table 3 and Figure 6. Table 3 shows the number of exams in which the analysis changed from positive (MBF_stress_ < 2.3 mL⋅min^−1^⋅g^−1^) to negative (MBF_stress_ > 2.3 mL⋅min^−1^⋅g^−1^) or negative to positive after motion correction. Figure 6 presents scatter plots and Bland–Altman plots of territorial and global MBF_stress_ for pre-correction vs motion-corrected images. The results suggest that global MBF_stress_ is relatively unaffected by motion, as correction led to a change in reading in only two exams (2.5%). At a territorial level, motion correction altered the analysis results of three exams in LAD, seven exams in RCA, and three exams in LCx. The largest spread and widest limits of agreement in the Bland–Altman plots were observed in RCA. Overall, four exams changed from positive to negative (5%), while two exams changed from negative to positive post-motion correction (2.5%) based on MBF in any of the territories in Table 3.

**Table 3:** The impact of motion correction on exam interpretation in 80 clinical exams, based on a 2.3 mL⋅min^−1^⋅g^−1^ threshold for MBF_stress_

	Global	LAD	RCA	LCX	Any
Positive to negative	1	0	4	1	4
Negative to positive	1	3	3	2	2

Exams were considered positive if MBF_stress_ < 2.3 mL⋅min^−1^⋅g^−1^ globally or in one or more coronary territories. Based on changes in MBF globally or in any of the coronary territories, motion correction altered the interpretation of six exams

## Discussion

In the first part of this study, we investigated the ability to visually assess and manually correct patient motion in 160 dynamic ^15^O-water PET scans with simulated motion mixed with 10 motion-free scans. All data were analyzed using two different motion correction approaches. Motion had a significant effect on MBF, as illustrated by the heat maps in Figure 2. The patterns of MBF deviations with negative median relative deviations in RCA and the largest deviations in LAD agreed with the previous study by Nordström et al.^6^

### The post-analysis approach led to fewer corrections

The motion correction problem consists of two parts: (1) Identifying significant motion and (2) correcting the motion. In the simulation study, despite a high prevalence of motion (94%), only 63 scans (37%) exhibited significant motion artifacts on the coronary territory level before correction. When using the pre-analysis approach, without any knowledge of the pre-correction results, nearly all scans (94%) were motion-corrected. However, when the post-analysis approach was used, in which the pre-correction results were reviewed, the number of corrected scans was only 64%. Many scans with motion were not corrected using the post-analysis approach, implying that scans without perceived motion artifacts in parametric images were left uncorrected.

### Large corrections performed mainly in the axial direction

The simulated data included motion isolated to the *y*-direction in 35%, to the *z*-direction in 35%, and in both the *y*- and *z*-direction in 24% of the scans and no motion in the *x*-direction. Yet, when using the pre-analysis approach, corrections were applied in the *x*-direction in 32% of the scans (in 8% > 10 mm), suggesting that visually identifying the direction of motion was difficult. When using the post-analysis approach, correction in the *x*-direction was applied to only one scan, however, this may be due to observer variation rather than the approach. Despite an equal distribution of simulated motion between the *y*- and *z*-directions, corrections were mainly applied in the *z*-direction for both approaches (44% in *y* vs 89% in *z* and 28% in *y* vs 44% in *z*, respectively). Similarly, large corrections (> 10 mm) were mainly applied in the *z*-direction for both approaches (25% in *y* vs 65% in *z* and 14% in *y* vs 28% in *z*, respectively), indicating that motion in the *z*-direction was more easily detectable. Both image readers were generally too conservative when it came to the magnitude of corrections and residual motion was found in most scans after correction (Figure 3). Both approaches tended to undercorrect more types of motion in the *y*- and *z*-directions and overcorrect fewer types of motion. The undercorrection was observed for the same types of motion regardless of the approach used.

### Large artifacts were reduced

**Figure 4:**
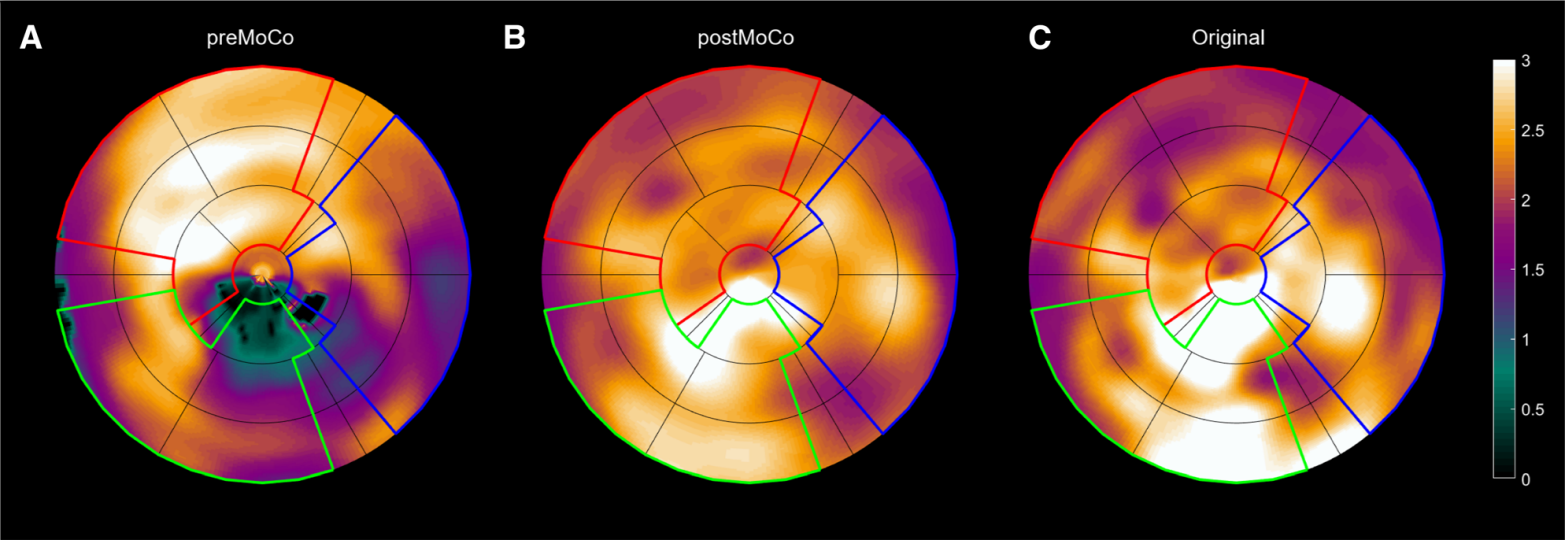
Polar plots from a patient scan comparing images pre-correction (**A**), post-correction (**B**), and original motion-free (**C**). The simulated linear slide motion (20 mm, 1 minute post-injection) increased MBF in the anterior part and reduced MBF in the inferior part with a large apical inferolateral defect (**A**). After motion correction, the artifact disappeared (**B**), aligning with the original motion-free image (**C**)

For large motion artifacts, both approaches yielded similar results as artifacts in 84% and 82% of the scans were reduced, respectively. Out of six scans with very large artifacts (> 80%), the motion artifact was reduced in five for both approaches. The reduction of motion-induced artifacts does not imply complete elimination of the artifact. In fact, the median reduction was only 46%, implying significant residual artifact in most scans. An example of successful correction of a large motion artifact is shown in Figure 4.

### Pattern of deviation in RCA and LAD persisted after correction

The impact of motion correction on median MBF deviations is visualized in Figure 2, showing a reduction in deviations after correction (Figure 2A–C) as expected. However, significant deviations in MBF persisted in most motion types, and the pattern of negative deviations in RCA and positive deviations in LAD remained. As for the large artifacts, motion correction could only reduce and not completely remove motion artifacts. Significant MBF deviations introduced by the motion correction were induced in three cells using the pre-analysis approach (Figure 2H). Two of the new deviations were in LAD and all in scans with motion in the anterior (+ *y*) and/or cranial direction (+ *z*). They were, however, negligible with median deviations below 5%. Even though no new significant deviations were introduced by using the post-analysis approach, it proved to be slightly less consistent in reducing or removing artifacts in RCA, caused by discomfort to the stress agent (Figure 2I) compared to the pre-analysis approach. This suggest that these artifacts were difficult to identify in some of the post-analysis parametric images. Nevertheless, median and maximum deviations were similar for both approaches in the RCA.

### Care should be taken when using hard cut-offs in cases with motion

Based on a MBF cut-off at 2.3 mL⋅min^−1^⋅g^−1^, 60% of the original motion-free scans were positive for reduced MBF_stress_ globally or in one or more coronary territory. This is higher than to be expected in a clinical cohort.^2^ In addition, median MBF of the original motion-free scans globally and in all territories were close to 2.3 mL⋅min^−1^⋅g^−1^. As an example, one original scan had a global MBF of 2.31 mL⋅min^−1^⋅g^−1^, and since it was represented 17 times, it resulted in 17 scans that could potentially become positive by a small reduction in MBF of only 0.02 mL⋅min^−1^⋅g^−1^. As a consequence and despite generally reduced motion artifacts, when applying either the pre- and post-analysis approach, half of the scans that underwent interpretation changes following motion correction in this study were incorrectly reclassified (Table 2). However, while the number of changes in interpretation may seem disconcerting, the absolute differences in MBF values were, as mentioned above, small.

In future studies, it may be useful to investigate the impact of patient motion on MBF in both high-and low-risk populations to better understand the potential impact of patient motion on clinical decision making.

### Post-analysis approach is the most feasible implementation

Overall, the impact of the pre- and post-analysis approaches were similar and large artifacts were identified and reduced to a similar extend. However, the post-analysis approach resulted in a large reduction in number of scans with applied correction. By adopting this approach, the potential benefits of motion correction can be achieved without unnecessarily intervening in motion-free scans, making it the most feasible implementation in a clinical setting.

### Reported number of motion cases in the clinical cohort was low

In the second part of the study, the clinical effect of motion correction was assessed in a retrospective review of 1545 clinical patient exams. Fifty exams (3%) were reported as ‘conclusive with reservations due to motion’ and only 14 (1%) exams were categorized as ‘inconclusive due to motion.’ Combining these two categories, the prevalence of motion in the current study was 4%, which is relatively low, as several groups reported a prevalence of 30-68%.^6–10,17^ Multiple factors play into these discrepancies. The current study was retrospective, and readers were not instructed to report all motion. Instead, motion was only reported if it was expected to have consequences for the reading and small artifacts or artifacts that would not influence the interpretation of the exams were ignored. This contrasts with studies where specific tests for motion were performed on all scans resulting in a much higher prevalence.

Interestingly, when the motion correction tool was provided, 80 out of the 762 exams were motion corrected, corresponding to a prevalence of 10%. This points to motion being more prevalent than indicated in the clinical exam reports, but also suggests that the readers were not certain about the significance of motion. Of the 80 clinical patient scans where motion correction was performed, MBF_stress_ below the threshold of normal myocardial perfusion of 2.3 mL⋅min^−1^⋅g^−1^ before motion correction in 44% on the global level and in 59% on the territorial level. In scans with reduced MBF_stress_ in only one coronary territory, the reduction was predominantly in RCA, which aligns with previous findings from simulation studies and other research that have shown that motion affects MBF particularly in RCA.^7,8,17^

### Motion correction in the clinical cohort had little impact on interpretation

**Figure 5:**
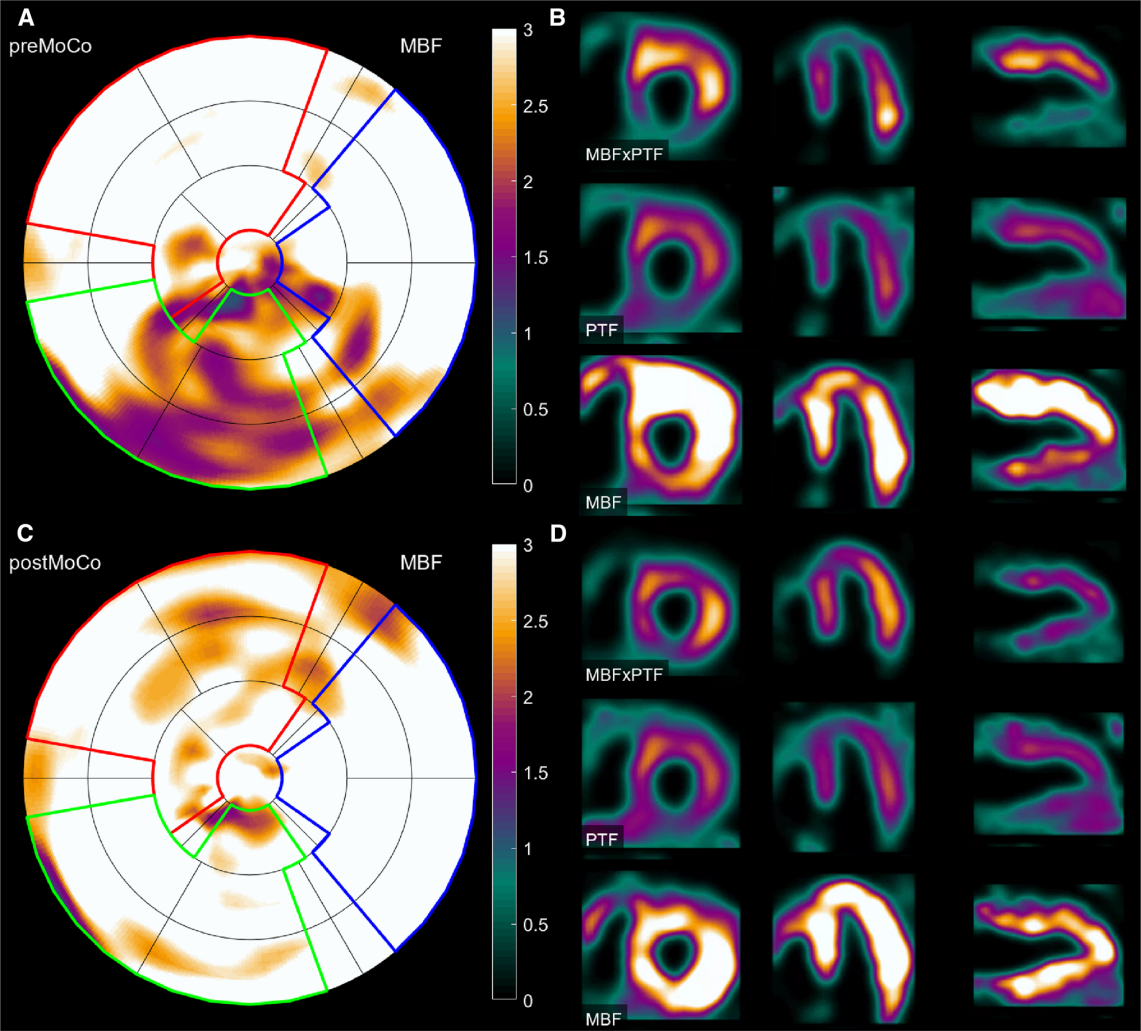
PET images representing a patient from the clinical cohort pre- (**A**, **B**) and post-motion (**C**, **D**) correction. In the polar plot in **A**, a motion artifact has caused a false positive defect (20.1 %) in the RCA territory. In the post-motion correction polar plot in **C**, the motion artifact is completely reduced (defect 0.0 %). Splash images of short axis, horizontal long axis, and vertical long axis in B demonstrate the effect of motion artifacts on the visual interpretation of the images. The inferior wall appears hypoperfused compared to the rest of the myocardium. The post-motion correction splash images in **D**, demonstrate a uniform tracer uptake, thereby eliminating any suspicions of defects in the inferior wall. *MBF*, myocardial blood flow (mL⋅min^−1^⋅g^−1^); *PTF*, perfusable tissue fraction (mL⋅mL^−1^)

**Figure 6:**
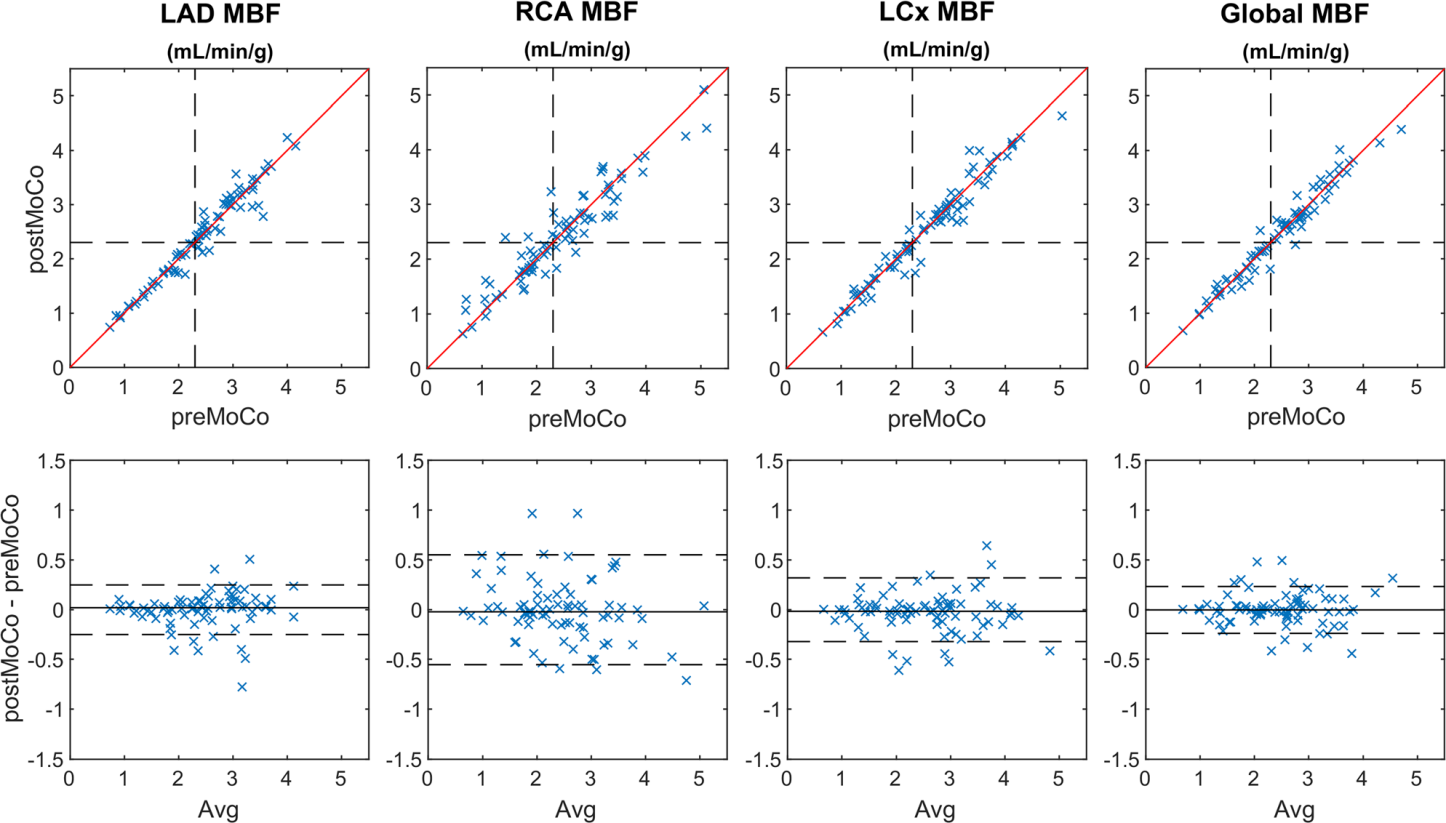
Scatter plots and Bland–Altman plots of regional MBFstress as well as global MBFstress for precorrection vs motion-corrected images in the clinical patient cohort. Dashed lines in the scatter plots indicate the threshold of normal values (MBF ≥ 2.3 mL⋅min^−1^⋅g^−1^). Red lines represent lines of identity. Dashed lines in the Bland–Altman plots represent limits of agreement

A clinical patient exam is displayed in Figure 5, illustrating the commonly observed motion artifact pattern with apparent anterior hyperperfusion and wall thickening combined with inferior hypoperfusion and wall thinning, found in 16% of exams. As shown in the simulation study, a range of stress agent responses and linear slide motions give rise to this artifact pattern. Pre-correction, the RCA defect was 20% of the LV, but after motion correction, it was eliminated. In most of the clinical cases (83.8%), motion was identified and corrected in the caudal (− *z*) or cranial (+ *z*) direction. This aligns with previous research reporting a frequent occurrence of patient motion in the *z*-direction,^9,23^ but may also reflect that motion in the *z*-direction is more easily identified compared to *x* and *y* motion as seen in the simulation study (Figure 6).

Motion correction had minimal impact on the interpretation of most exams. Only five of 240 coronary territories changed from positive (< 2.3 mL⋅min^−1^⋅g^−1^) to negative (≥ 2.3 mL⋅min^−1^⋅g^−1^) and eight changed from negative to positive after motion correction. However, some changes were in the same exam and, in only six exams, readings changed from hypoperfusion in one or more coronary territories to normal perfusion in all territories (n = 4) or vice a versa (n = 2). An additional four exams had an increase in the number of territories with hypoperfusion corresponding to a total impact of the correction in 12.5% of the motion corrected exams or 1% of the entire clinical patient cohort.

## Limitations

The simulated data exhibited an artificially high occurrence of motion (160 out of 170 scans). Despite this, as demonstrated by Nordström et al.,^6^ most of the scans did not display any significant motion artifacts.

With frame-to-frame motion correction, only inter-frame motion can be corrected, without the possibility to assess intra-frame motion. In the simulated data, the applied motion types were all inter-frame motion as well. Often, patient motion is seen as intra-frame motion, which is difficult to detect and correct.

## New knowledge gained

Manual motion correction in ^15^O-water cardiac PET reduces but rarely eliminates motion artifacts. Identifying motion axis and magnitude through visual assessment of dynamic frames is challenging, leading to primarily axial correction and generally undercorrection. Pre-correction review of MBF parametric images reduces unnecessary corrections and may be the most practical implementation. In a clinical cohort, manual motion correction affected exam result interpretation in only 12.5% of cases.

## Conclusion

Manual frame-by-frame motion correction after visual inspection is efficient in reducing but not removing motion artifacts in cardiac ^15^O-water PET. Utilizing pre-correction results for the assessment of motion did not significantly aid in the identification of large motion but resulted in fewer corrections in motion-free scans. Few motion artifacts were aggravated by motion correction. In a large clinical cohort, the impact of motion correction was limited to few patients.

### Supplementary Information

Below is the link to the electronic supplementary material.

Supplementary file1 (DOCX 399 KB)

Supplementary file2 (PPTX 1346 KB)

Supplementary file3 (MP3 4370 KB)

## Abbreviations

CT: Computed tomography
LAD: Left anterior descending coronary artery
LCx: Left circumflex coronary artery
LV: Left ventricular
MBF: Myocardial blood flow
MPI: Myocardial perfusion imaging
PET: Positron emission tomography
RCA: Right coronary artery
