# Supplementary material for: Detection and correction of patient motion in dynamic 15O-water PET MPI
Source: J Nucl Cardiol. 2023 Aug 28;30(6):2736–49. doi: 10.1007/s12350-023-03358-5 (PMC10682105; doi:10.1007/s12350-023-03358-5)

**Supplementary material - Detection and Correction of Patient Motion in Dynamic ^15^O-water PET MPI**

Nana L. Christensen, Jonny Nordström, Simon Madsen, Michael A. Madsen, Lars C. Gormsen, Tanja Kero, Mark Lubberink and Lars P. Tolbod

**Table S1:** Summary of simulated motions. (*) not included in Nordström et al (ref 6)

| **Motion** | **Type** | **Direction** | **Simulation** |
| --- | --- | --- | --- |
| No motion | None | None | None |
| Stress agent 1 | 1 | +y and +z | $2*e^{- \frac{\log2*t}{0.2}}$ |
| Stress agent 2 | 1 | +y and +z | $1*e^{- \frac{\log2*t}{0.2}}$ |
| Stress agent 3 | 1 | +y and +z | $2*e^{- \frac{\log2*t}{0.5}}$ |
| Stress agent 4 | 1 | +y and +z | $1*e^{- \frac{\log2*t}{0.5}}$ |
| Linear slide 1 | 2 | -z | 10 mm linear slide in caudal direction during whole scan |
| Linear slide 2 | 2 | -z | 20 mm linear slide in caudal direction during whole scan |
| Linear slide 3* | 2 | -z | 10 mm linear slide in caudal direction after 1 min post injection to end scan |
| Linear slide 4* | 2 | -z | 20 mm linear slide in caudal direction after 1 min post injection to end scan |
| Peak cough 1 | 3 | +y | 10 mm single frame anterior displacement at frame of peak myocardial TAC |
| Peak cough 2 | 3 | +y | 20 mm single frame anterior displacement at frame of peak myocardial TAC |
| Cardiac creep 1 | 4 | +z | 5 mm linear slide in cranial direction from 1 min post injection to end scan |
| Cardiac creep 2 | 4 | +z | 10 mm linear slide in cranial direction from 1 min post injection to end scan |
| Late cough 1 | 3 | +y | 20 mm single frame anterior displacement after 1 min |
| Late cough 2 | 3 | +y | 10 mm single frame anterior displacement after 1 min |
| Late cough 3* | 3 | +y | 20 mm single frame anterior displacement after 2 min |
| Late cough 4* | 3 | +y | 10 mm single frame anterior displacement after 2 min |

**Figure S1:** Heat maps comparing MBF deviations above or below the threshold of 2.3 mL/min/g in the original scan (A) and subsequent improvement achieved through motion correction using the pre-analysis (B) and post-analysis (C) approaches.


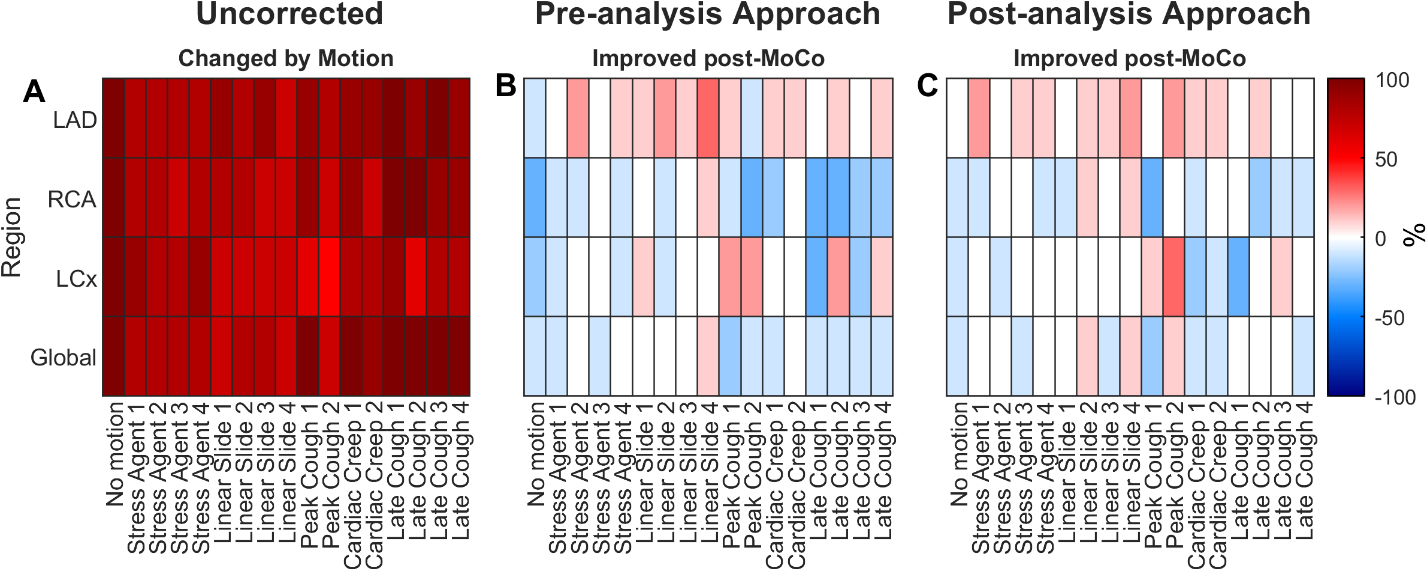

Supplement: Supplementary file 1 — Supplementary file1 (DOCX 399 KB) [file 12350_2023_3358_MOESM1_ESM.docx]
