# Supplementary material for: Detection and correction of patient motion in dynamic 15O-water PET MPI
Source: J Nucl Cardiol. 2023 Aug 28;30(6):2736–49. doi: 10.1007/s12350-023-03358-5 (PMC10682105; doi:10.1007/s12350-023-03358-5)
Supplement: Supplementary file 2 — Supplementary file2 (PPTX 1346 KB) [file 12350_2023_3358_MOESM2_ESM.pptx]

## Slide 1
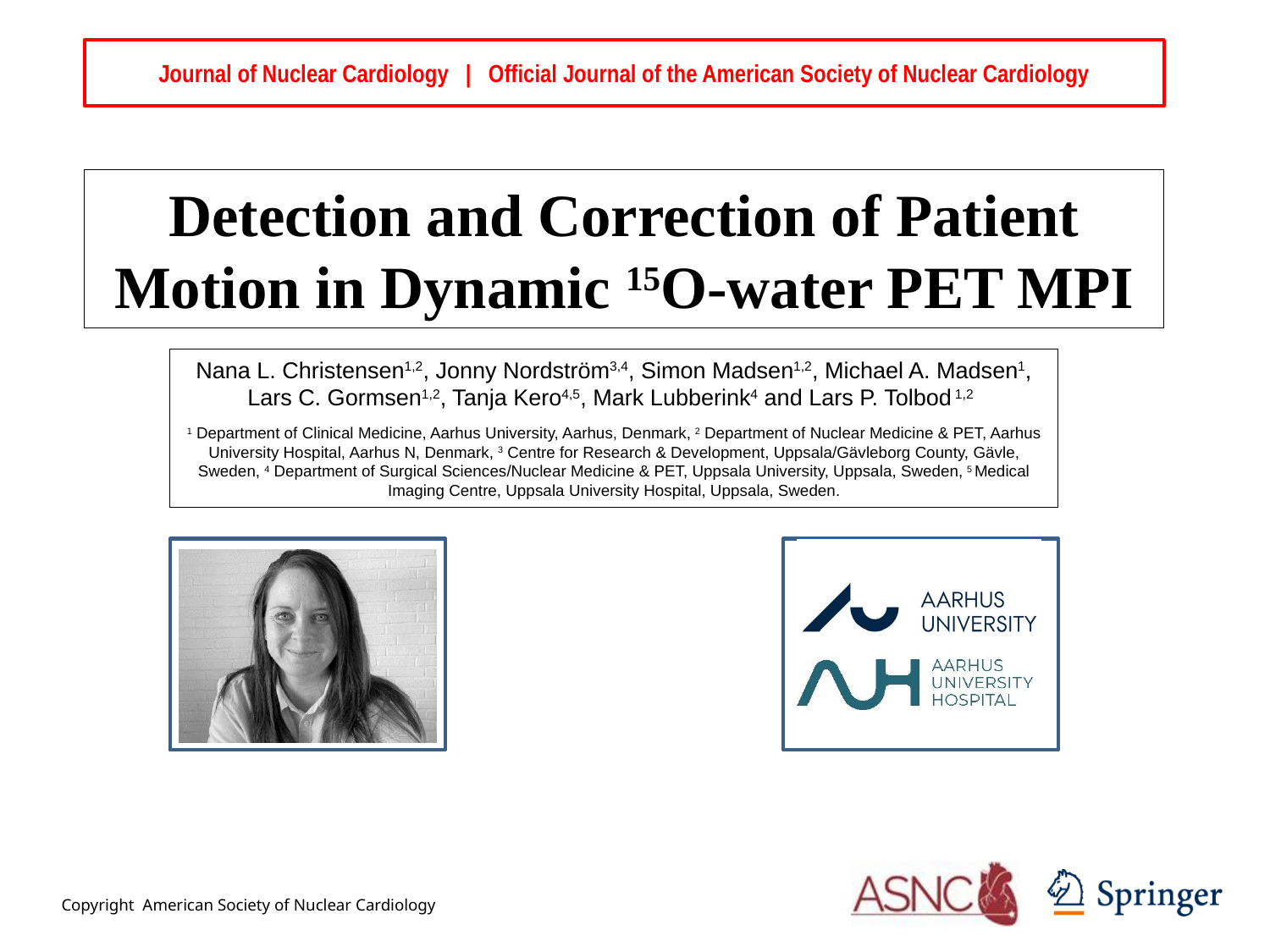

Journal of Nuclear Cardiology | Official Journal of the American Society of Nuclear Cardiology
# Detection and Correction of Patient Motion in Dynamic 15O-water PET MPI
Nana L. Christensen1,2, Jonny Nordström3,4, Simon Madsen1,2, Michael A. Madsen1, Lars C. Gormsen1,2, Tanja Kero4,5, Mark Lubberink4 and Lars P. Tolbod 1,2
1 Department of Clinical Medicine, Aarhus University, Aarhus, Denmark, 2 Department of Nuclear Medicine & PET, Aarhus University Hospital, Aarhus N, Denmark, 3 Centre for Research & Development, Uppsala/Gävleborg County, Gävle, Sweden, 4 Department of Surgical Sciences/Nuclear Medicine & PET, Uppsala University, Uppsala, Sweden, 5 Medical Imaging Centre, Uppsala University Hospital, Uppsala, Sweden.
Head shot of author
required
Copyright American Society of Nuclear Cardiology

## Slide 2
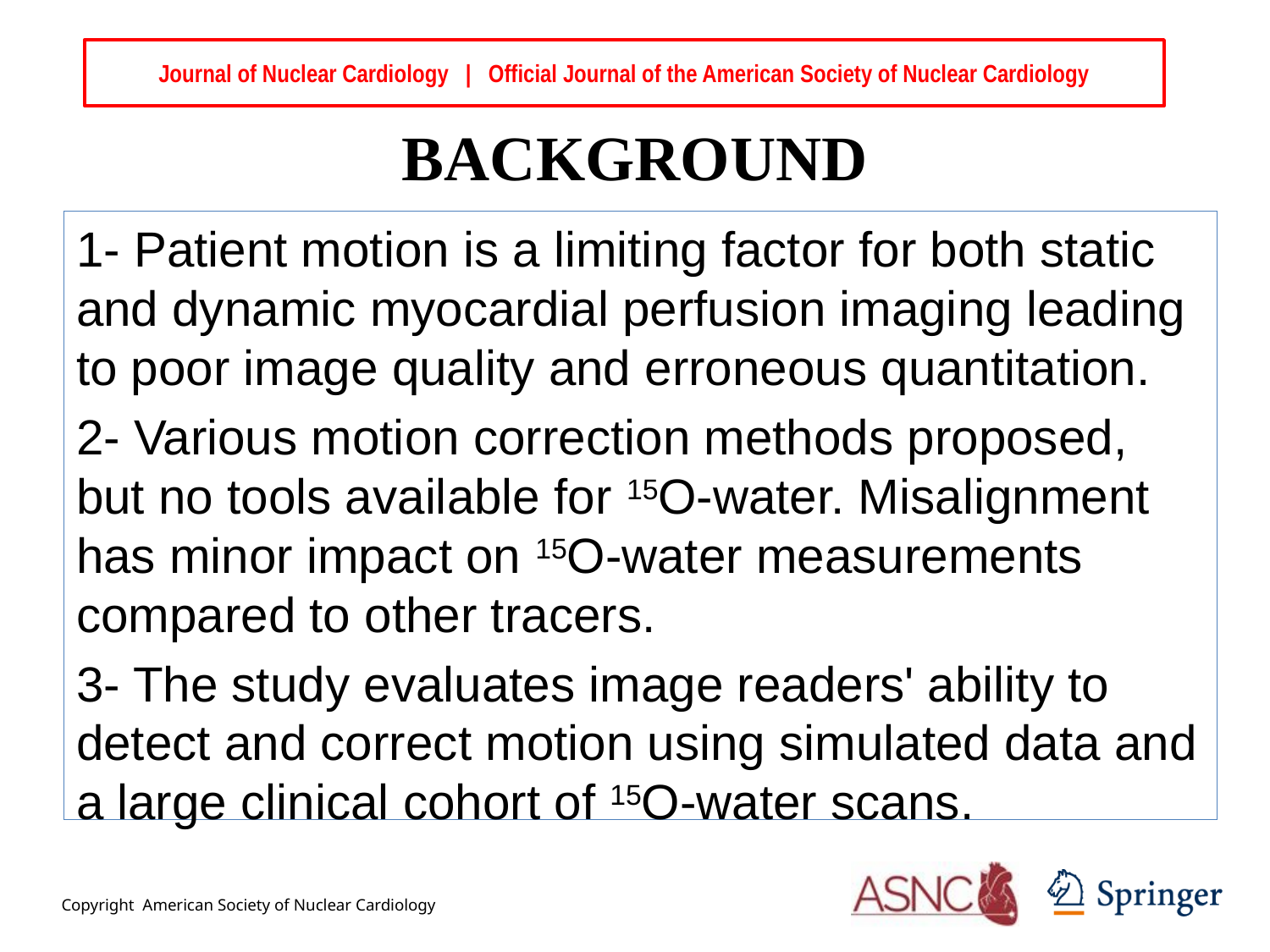

Journal of Nuclear Cardiology | Official Journal of the American Society of Nuclear Cardiology
# BACKGROUND
1- Patient motion is a limiting factor for both static and dynamic myocardial perfusion imaging leading to poor image quality and erroneous quantitation.
2- Various motion correction methods proposed, but no tools available for 15O-water. Misalignment has minor impact on 15O-water measurements compared to other tracers.
3- The study evaluates image readers' ability to detect and correct motion using simulated data and a large clinical cohort of 15O-water scans.
Copyright American Society of Nuclear Cardiology

## Slide 3
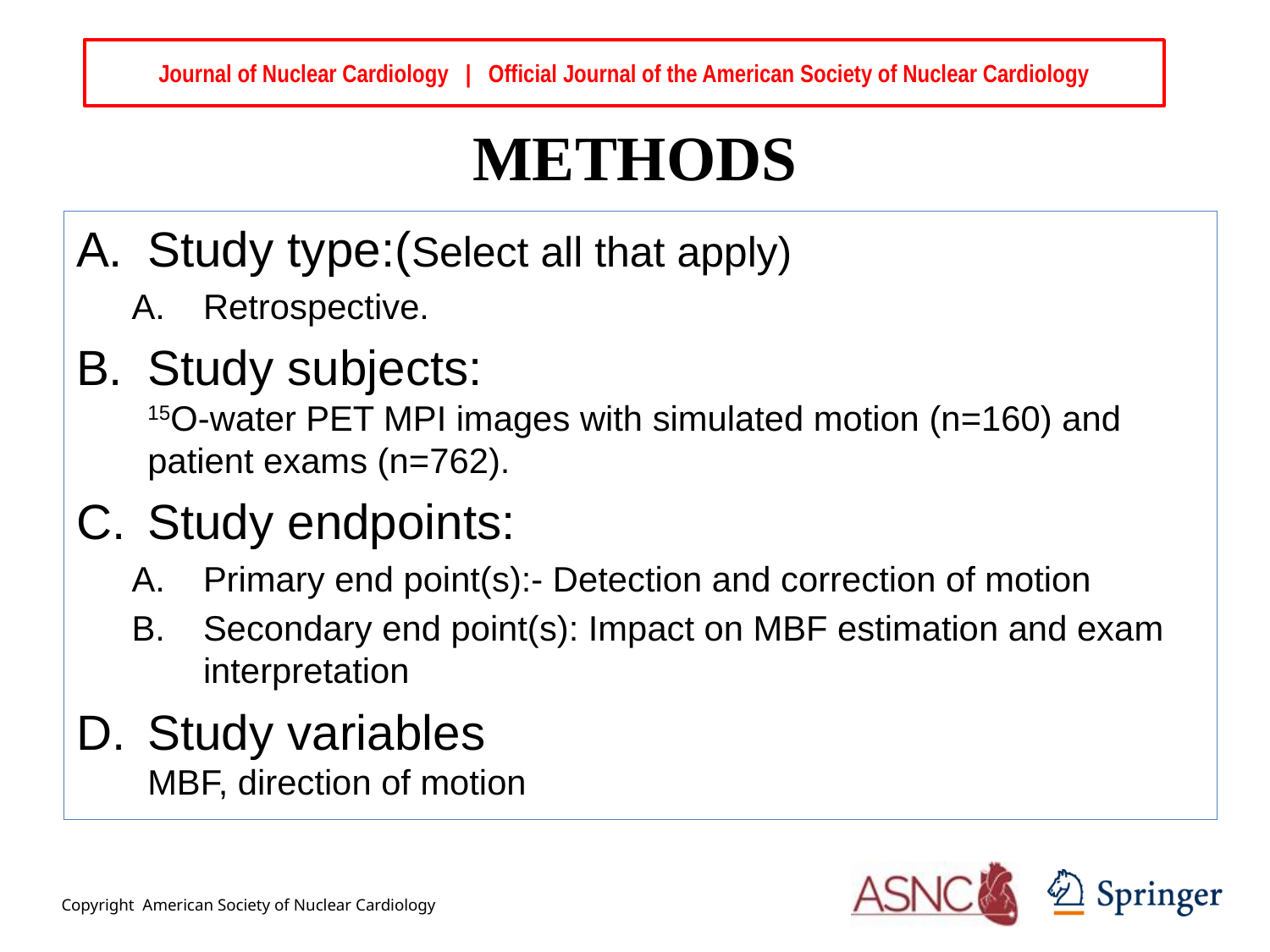

Journal of Nuclear Cardiology | Official Journal of the American Society of Nuclear Cardiology
# METHODS
Study type:(Select all that apply)
Retrospective.
Study subjects:
15O-water PET MPI images with simulated motion (n=160) and patient exams (n=762).
Study endpoints:
Primary end point(s):- Detection and correction of motion
Secondary end point(s): Impact on MBF estimation and exam interpretation
Study variables
MBF, direction of motion
Copyright American Society of Nuclear Cardiology

## Slide 4
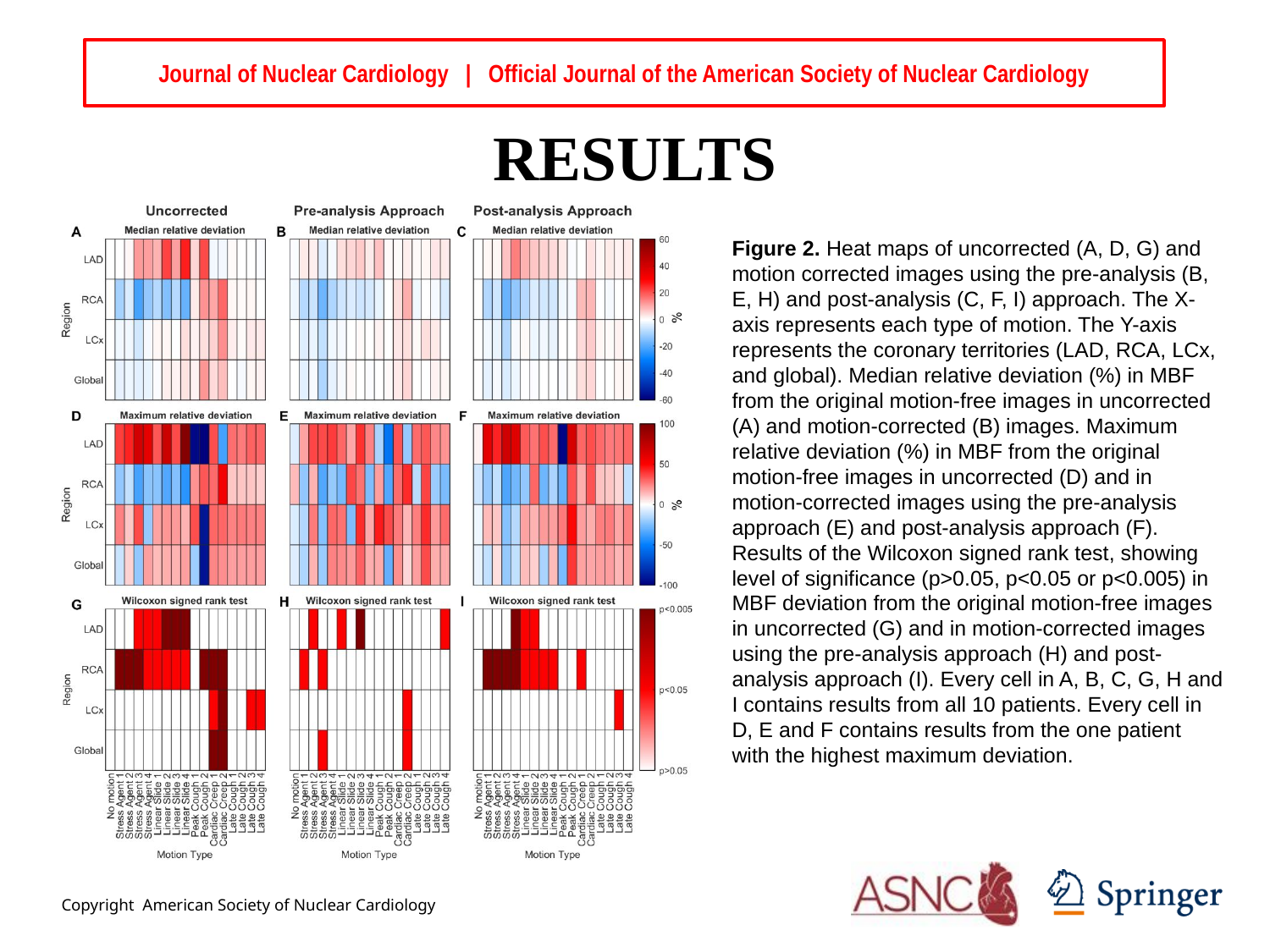

Journal of Nuclear Cardiology | Official Journal of the American Society of Nuclear Cardiology
# RESULTS
Figure 2. Heat maps of uncorrected (A, D, G) and motion corrected images using the pre-analysis (B, E, H) and post-analysis (C, F, I) approach. The X-axis represents each type of motion. The Y-axis represents the coronary territories (LAD, RCA, LCx, and global). Median relative deviation (%) in MBF from the original motion-free images in uncorrected (A) and motion-corrected (B) images. Maximum relative deviation (%) in MBF from the original motion-free images in uncorrected (D) and in motion-corrected images using the pre-analysis approach (E) and post-analysis approach (F). Results of the Wilcoxon signed rank test, showing level of significance (p>0.05, p<0.05 or p<0.005) in MBF deviation from the original motion-free images in uncorrected (G) and in motion-corrected images using the pre-analysis approach (H) and post-analysis approach (I). Every cell in A, B, C, G, H and I contains results from all 10 patients. Every cell in D, E and F contains results from the one patient with the highest maximum deviation.
Copyright American Society of Nuclear Cardiology

## Slide 5
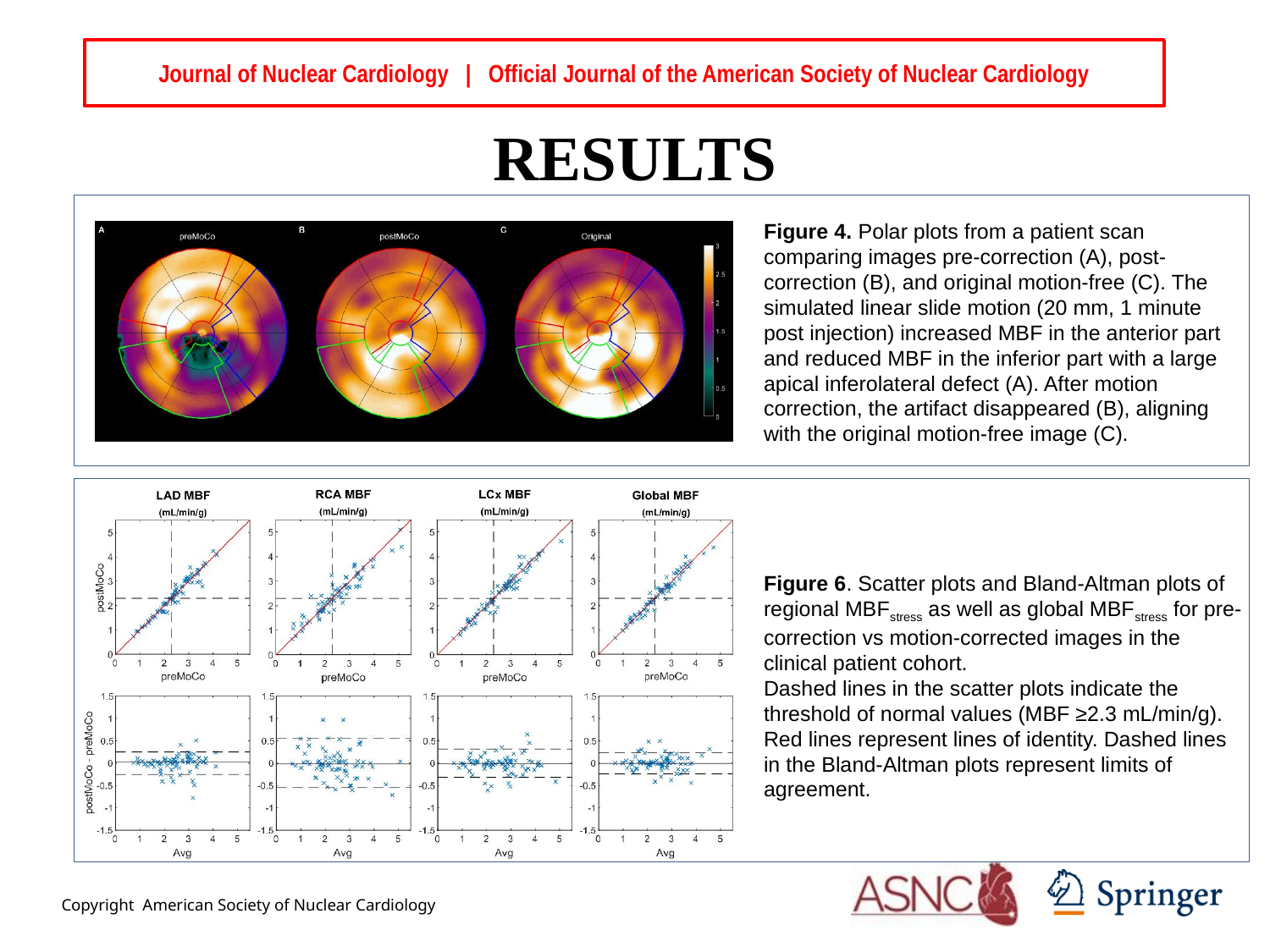

Journal of Nuclear Cardiology | Official Journal of the American Society of Nuclear Cardiology
# RESULTS
Figure 4. Polar plots from a patient scan comparing images pre-correction (A), post-correction (B), and original motion-free (C). The simulated linear slide motion (20 mm, 1 minute post injection) increased MBF in the anterior part and reduced MBF in the inferior part with a large apical inferolateral defect (A). After motion correction, the artifact disappeared (B), aligning with the original motion-free image (C).
Figure 6. Scatter plots and Bland-Altman plots of regional MBFstress as well as global MBFstress for pre-correction vs motion-corrected images in the clinical patient cohort.
Dashed lines in the scatter plots indicate the threshold of normal values (MBF ≥2.3 mL/min/g). Red lines represent lines of identity. Dashed lines in the Bland-Altman plots represent limits of agreement.
Copyright American Society of Nuclear Cardiology

## Slide 6
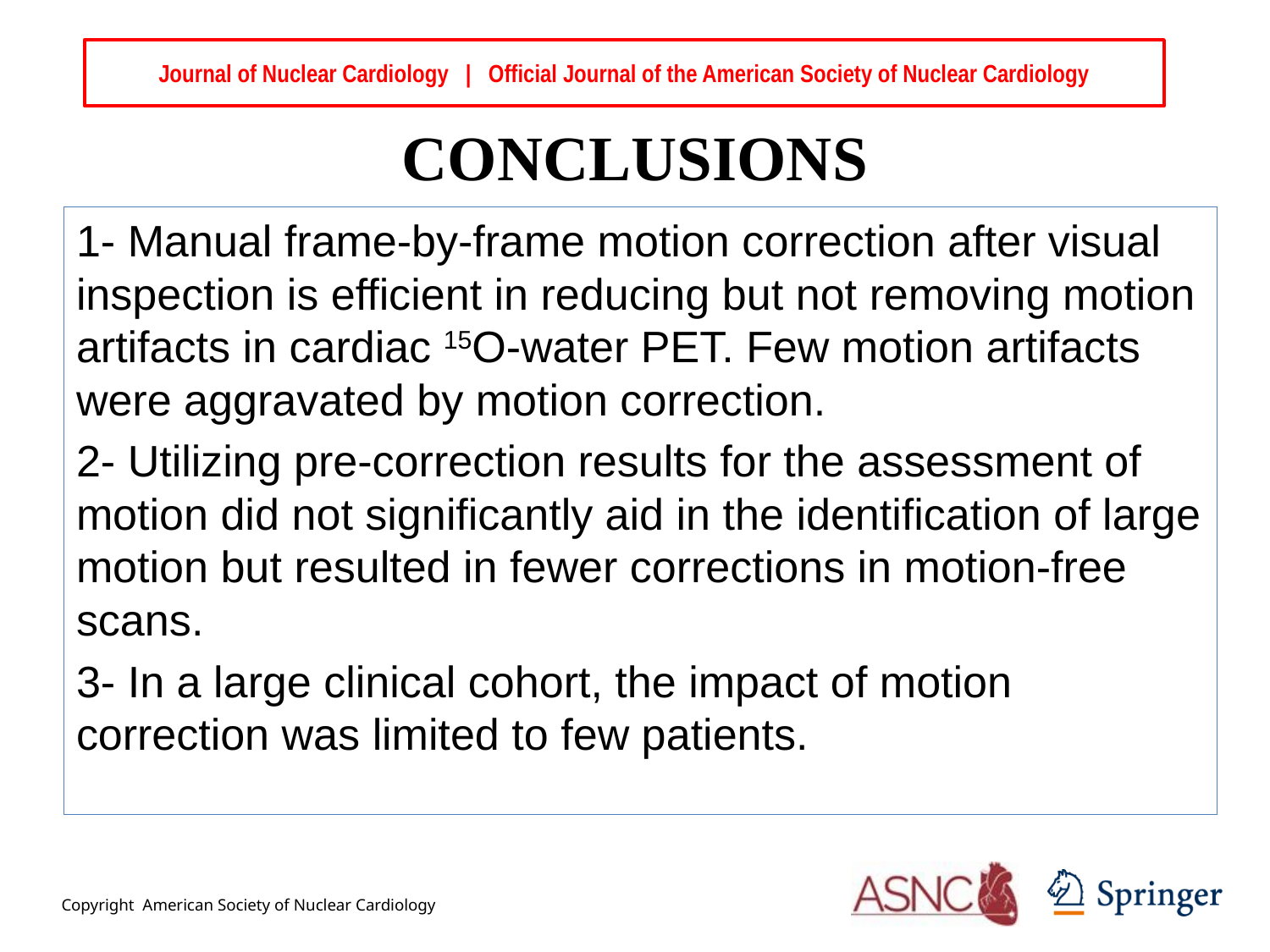

Journal of Nuclear Cardiology | Official Journal of the American Society of Nuclear Cardiology
# CONCLUSIONS
1- Manual frame-by-frame motion correction after visual inspection is efficient in reducing but not removing motion artifacts in cardiac 15O-water PET. Few motion artifacts were aggravated by motion correction.
2- Utilizing pre-correction results for the assessment of motion did not significantly aid in the identification of large motion but resulted in fewer corrections in motion-free scans.
3- In a large clinical cohort, the impact of motion correction was limited to few patients.
Copyright American Society of Nuclear Cardiology
